# Household and maternal risk factors for malaria in pregnancy in a highly endemic area of Uganda: a prospective cohort study

**DOI:** 10.1186/s12936-019-2779-x

**Published:** 2019-04-23

**Authors:** Jaffer Okiring, Peter Olwoch, Abel Kakuru, Joseph Okou, Harriet Ochokoru, Tedy Andra Ochieng, Richard Kajubi, Moses R. Kamya, Grant Dorsey, Lucy S. Tusting

**Affiliations:** 1 Infectious Diseases Research Collaboration, 2C Nakasero Hill Road, Kampala, Uganda; 2 Makerere University College of Health Sciences, Kampala, Uganda; 3 University of California, San Francisco, San Francisco, CA 94110 USA; 4 Department of Disease Control, London School of Hygiene & Tropical Medicine, Keppel Street, London, WC1E 7HT UK

**Keywords:** Malaria in pregnancy, Risk factors, Area of high malaria endemicity

## Abstract

**Background:**

Malaria in pregnancy is a major public health challenge, but its risk factors remain poorly understood in some settings. This study assessed the association between household and maternal characteristics and malaria among pregnant women in a high transmission area of Uganda.

**Methods:**

A nested prospective study was conducted between 6th September 2016 and 5th December 2017 in Busia district. 782 HIV uninfected women were enrolled in the parent study with convenience sampling. Socioeconomic and house construction data were collected via a household survey after enrolment. Homes were classified as modern (plaster or cement walls, metal or wooden roof and closed eaves) or traditional (all other homes). Maternal and household risk factors were evaluated for three outcomes: (1) malaria parasitaemia at enrolment, measured by thick blood smear and qPCR, (2) malaria parasitaemia during pregnancy following initiation of IPTp, measured by thick blood smear and qPCR and (3) placental malaria measured by histopathology.

**Results:**

A total of 753 of 782 women were included in the analysis. Most women had no or primary education (75%) and lived in traditional houses (77%). At enrolment, microscopic or sub-microscopic parasitaemia was associated with house type (traditional versus modern: adjusted risk ratio (aRR) 1.29, 95% confidence intervals 1.15–1.45, p < 0.001), level of education (primary or no education versus O-level or beyond: aRR 1.13, 95% confidence interval 1.02–1.24, p = 0.02), and gravidity (primigravida versus multigravida: aRR 1.10, 95% confidence interval 1.02–1.18, p = 0.009). After initiation of IPTp, microscopic or sub-microscopic parasitaemia was associated with wealth index (poorest versus least poor: aRR 1.24, 95% CI 1.10–1.39, p < 0.001), house type (aRR 1.14, 95% CI 1.01–1.28, p = 0.03), education level (aRR 1.19, 95% CI 1.06–1.34, p = 0.002) and gravidity (aRR 1.32, 95% CI 1.20–1.45, p < 0.001). Placental malaria was associated with gravidity (aRR 2.87, 95% CI 2.39–3.45, p < 0.001), but not with household characteristics.

**Conclusions:**

In an area of high malaria transmission, primigravid women and those belonging to the poorest households, living in traditional homes and with the least education had the greatest risk of malaria during pregnancy.

## Background

Malaria remains a major preventable cause of maternal morbidity and adverse birth outcomes in Africa, where an estimated 12.4 million pregnant women were exposed to malaria in 2010 [1]. Although most malaria infections during pregnancy remain asymptomatic in endemic areas, these infections are associated with maternal anaemia and poor birth outcomes including preterm birth, low birth weight (LBW) and perinatal mortality [2, 3]. For prevention of malaria in pregnancy the World Health Organization (WHO) recommends the use of long-lasting insecticide-treated nets (LLINs), intermittent preventive treatment (IPTp) with sulfadoxine–pyrimethamine (SP) and prompt diagnosis and effective case management. However, widespread parasite resistance to SP and mosquito resistance to the pyrethroids used in LLINs has led to concern over reduced efficacy of these interventions [4, 5]. Therefore, additional approaches to prevent malaria in pregnancy and improve birth outcomes are needed.

Social and environmental factors such as wealth [6] and house design [7] can be important determinants of malaria risk that may inform supplementary approaches to malaria control [8], but there are only a few examples of studies examining these risk factors in relation to pregnant women [9, 10]. Indeed, most observational studies of malaria in pregnancy have explored factors associated with uptake of anti-malarial interventions [11–16] and perceptions of malaria in pregnancy [17, 18], as well as maternal risk factors for malaria in pregnancy [10, 19, 20]. In Uganda, it has been observed that younger and less educated women are at greater risk of malaria in pregnancy [21], while IRS and ≥ 2 doses of SP during pregnancy may offer some protection against adverse birth and maternal outcomes [5, 22]. In this study, maternal and household risk factors for malaria were evaluated in a high malaria transmission setting in Busia, eastern Uganda. This study is one of the first to examine the association between household characteristics and malaria in pregnancy in Uganda.

## Methods

### Study setting and participants

This study was conducted in Busia district, an area in south-eastern Uganda where malaria transmission is perennial and holoendemic. This prospective cohort study was part of a randomized controlled trial of intermittent preventive treatment of malaria in pregnancy (IPTp), which has been previously described [23]. Briefly, eligible participants for the parent study were HIV-uninfected women at least 16 years of age with a viable pregnancy between 12 and 20 weeks gestation who provided written informed consent.

### Study procedures

At enrolment, women received a long-lasting insecticidal net (LLIN), underwent a standardized history and examination and had blood collected for the detection of malaria parasites by microscopy and quantitative PCR (qPCR). Women were randomized (1:1 ratio) to receive IPTp with monthly sulfadoxine–pyrimethamine (SP) or monthly dihydroartemisinin–piperaquine (DP) starting at 16 or 20 weeks gestational age as previously described [23]. Following enrolment, women were visited at home where a household survey was conducted to collect socioeconomic and house construction data using a structured questionnaire.

Women received all their medical care at a study clinic open every day. Routine visits at the study clinic were conducted every 4 weeks, including collection of blood for the detection of malaria parasites by microscopy and quantitative qPCR. Women were encouraged to come to the clinic any time they were ill. Those who presented with a documented fever (tympanic temperature ≥ 38.0 °C) or history of fever in the previous 24 h had blood collected for a thick blood smear. If the smear was positive, the patient was diagnosed with malaria and treated with artemether–lumefantrine. Women were encouraged to deliver at the hospital adjacent to the study clinic. Women delivering at home were visited by study staff at the time of delivery or as soon as possible afterwards. At delivery, a standardized assessment was completed including collection of placental tissue for assessment of placental malaria.

### Laboratory procedures

Blood smears were stained with 2% Giemsa and read by experienced microscopists. A blood smear was considered negative when the examination of 100 high power fields did not reveal asexual parasites. For quality control, all slides were read by a second microscopist and a third reviewer settled any discrepant readings. Blood samples collected at enrolment and at the time of each routine visit that were negative by microscopy were tested for the presence of submicroscopic parasitaemia using a highly sensitive qPCR assay targeting the multicopy conserved *var* gene acidic terminal sequence with a lower limit of detection of 1 parasite/ml [24]. Placental tissues were processed for histological evidence of placental malaria as previously described [23].

### Data management and statistical analysis

Data were collected in the study clinic using standardized case record forms entered into Microsoft Access. Data from the household survey were collected using hand-held computers and customized software designed and programmed to include range checks and internal consistency checks. All statistical analyses were performed using Stata version 14.1 (StataCorp, College Station, TX, USA).

Exposure variables of interest included characteristics of the study participants (education, bed net ownership, gravidity and IPTp regimen) and their households (wealth index and house construction). Principal component analysis was used to generate a wealth index based on ownership of common household items. Households were ranked by wealth scores and grouped into tertiles to give a categorical measure of socioeconomic position. House types were classified based on definitions previously developed for the study area [25]. Modern houses were defined as having plaster or cement walls, metal or wooden roofs, and closed eaves; all other houses were defined as traditional. Three outcome measures were assessed: (1) microscopic and microscopic or sub-microscopic parasitaemia at enrolment, (2) microscopic and microscopic or sub-microscopic parasitaemia at the time of routine visits during pregnancy following initiation of IPTp, and (3) placental malaria based on the detection of malaria parasites or pigment by histopathology. Associations between exposure variables and parasitaemia at enrolment or placental malaria were estimated using generalized linear models with a Poisson family and robust error variance. Associations between exposure variables and parasitaemia during pregnancy were estimated using generalized estimating equations to adjust for repeated measures in the same study participant with a Poisson family and robust error variance. Measures of association were expressed as unadjusted and adjusted relative risks (RR and aRR, respectively) and p-values (two-sided) < 0.05 were considered statistically significant.

## Results

### Characteristics of participants and their households

**Fig. 1:**
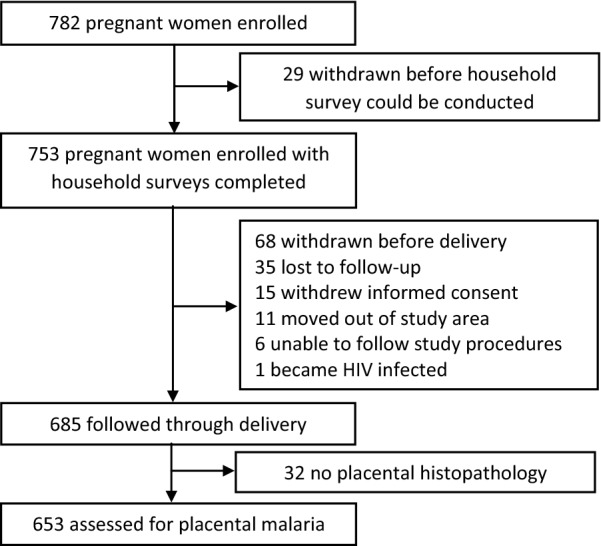
Trial profile

Among 782 women enrolled in the parent study, 29 were withdrawn before a household survey could be completed resulting in 753 women included in the assessment of parasitaemia at enrolment and during pregnancy (Fig. 1). Most women lived in houses constructed using traditional materials (77.2%), with no airbricks (72.2%) and at least one window (78.5%) (Table 1). Most women were not educated beyond primary school (75.3%) but a majority reported owning an LLIN before enrolment (76.9%). Approximately half the women were multigravidae (at least 2 prior pregnancies) and assigned IPTp regimens were equally distributed as expected. Among women with household surveys completed, 68 were withdrawn before delivery and 32 had no placental tissue collected, resulting in 653 women included in the assessment of placental malaria (Fig. 1).

**Table 1:** Characteristics of study participants

Variable	Categories	Wealth index category				
		All (n = 753)	Least poor (n = 244)	Middle (n = 252)	Poorest (n = 257)	p
Household characteristics (%)						
Type of house construction^a^	Modern^a^	22.8	38.1	13.1	17.9	< 0.001
	Traditional	77.2	61.9	86.9	82.1	
Whether airbricks were present	Present	27.8	45.1	18.7	20.2	< 0.001
	Not present	72.2	54.9	81.4	79.8	
Number of windows present	≥ 2 windows	19.1	32.0	13.9	12.1	< 0.001
	1 window	59.4	57.0	59.1	61.9	
	No windows	21.5	11.1	27.0	26.1	
Maternal characteristics (%)						
Level of education	O level or beyond	24.7	39.3	18.7	16.7	< 0.001
	None or primary	75.3	60.7	81.4	83.3	
Bed net ownership before enrolment	LLIN	76.9	84.0	78.2	68.9	0.001
	Untreated	14.5	11.1	14.7	17.5	
	No bed net	8.6	4.9	7.1	13.6	
Categories of gravidity	Multigravida	50.7	54.5	51.6	46.3	0.16
	Secundigravida	24.8	25.8	21.8	26.9	
	Primigravida	24.4	19.7	26.6	26.9	
IPTp regimen	Monthly DP	50.1	46.7	52.8	50.6	0.39
	Monthly SP	49.9	53.3	47.2	49.4	

^a^Modern houses defined as those with plaster or cement walls, metal or wooden roof, and closed eaves; all other houses defined as traditional

### Factors associated with parasitaemia at enrolment

At enrolment, 51.1% of women had malaria parasites detected by microscopy and 81.8% had malaria parasites detected by microscopy or qPCR. In multivariate analysis, women in the poorest households had a 29% greater risk of microscopic parasitaemia compared to the least poor (adjusted Risk Ratio (aRR) 1.29, 95% confidence intervals (CI) 1.07–1.55, p = 0.008). Similarly, women living in houses with traditional construction had a 41% greater risk of microscopic parasitaemia compared to women living in houses with modern construction (aRR = 1.41, 95% CI 1.14–1.74, p = 0.002). The strongest risk factor for microscopic parasitaemia at enrolment was gravidity, with primigravid women having almost twice the risk compared to multigravid women (aRR = 1.84, 95% CI 1.58–2.16, p < 0.001). The presence of airbricks or windows, level of education, and bed net ownership were not associated with microscopic parasitaemia at enrolment in multivariate analyses (Table 2). Results were similar but less pronounced for microscopic or sub-microscopic parasitaemia at enrolment, although a lower level of education (none or primary) was also associated with an increased risk of parasitaemia in multivariate analysis, compared to more education (O level or beyond) (aRR = 1.13, 95% CI 1.02–1.24, p = 0.02) (Table 3).

**Table 2:** Factors associated with microscopic parasitaemia at enrolment

Variable	Categories	Parasitaemia n (%)	Univariate analysis		Multivariate analysis	
			RR (95% CI)	p-value	aRR^a^ (95% CI)	p-value
Wealth index categories	Least poor	98 (40.2)	Reference	–	Reference	–
	Middle	133 (52.8)	1.31 (1.08–1.59)	0.005	1.15 (0.95–1.39)	0.15
	Poorest	154 (59.9)	1.49 (1.24–1.79)	< 0.001	1.29 (1.07–1.55)	0.008
Type of house construction^b^	Modern	61 (35.5)	Reference	–	Reference	–
	Traditional	324 (55.8)	1.57 (1.27–1.95)	< 0.001	1.41 (1.14–1.74)	0.002
Whether airbricks were present	Present	79 (37.8)	Reference	–	Reference	–
	Not present	306 (56.3)	1.49 (1.23–1.80)	< 0.001	1.09 (0.86–1.39)	0.48
Number of windows present	≥ 2 windows	65 (45.1)	Reference	–	Reference	–
	1 window	224 (50.1)	1.11 (0.91–1.36)	0.31	0.97 (0.79–1.18)	0.73
	No windows	96 (59.3)	1.31 (1.05–1.64)	0.02	1.06 (0.84–1.32)	0.63
Level of education	O level or beyond	86 (46.2)	Reference	–	Reference	–
	None or primary	299 (52.7)	1.14 (0.96–1.36)	0.14	1.13 (0.95–1.34)	0.17
Bed net ownership before enrolment	LLIN	285 (49.2)	Reference	–	Reference	–
	Untreated	62 (56.9)	1.16 (0.96–1.39)	0.12	1.07 (0.90–1.28)	0.45
	No bed net	38 (58.5)	1.19 (0.95–1.48)	0.13	1.03 (0.83–1.28)	0.77
Categories of gravidity	Multigravida	145 (38.0)	Reference	–	Reference	–
	Secundigravida	108 (57.8)	1.52 (1.27–1.82)	< 0.001	1.53 (1.28–1.82)	< 0.001
	Primigravida	132 (71.7)	1.89 (1.62–2.21)	< 0.001	1.84 (1.58–2.16)	< 0.001

*RR* risk ratio

^a^Adjusted for wealth index, type of house construction, level of education, bed net ownership before enrolment and categories of gravidity

^b^Modern houses defined as those with plaster or cement walls, metal or wooden roof, and closed eaves; all other houses defined as traditional

**Table 3:** Factors associated with microscopic or sub-microscopic parasitaemia at enrolment

Variable	Categories	Parasitaemia n (%)	Univariate analysis		Multivariate analysis	
			RR (95% CI)	p-value	aRR^a^ (95% CI)	p-value
Wealth index categories	Least poor	179 (73.4)	Reference	–	Reference	–
	Middle	215 (85.3)	1.16 (1.06–1.27)	0.001	1.07 (0.98–1.16)	0.16
	Poorest	222 (86.4)	1.18 (1.08–1.29)	< 0.001	1.09 (0.99–1.19)	0.07
Type of house construction^b^	Modern	111 (64.5)	Reference	–	Reference	–
	Traditional	505 (86.9)	1.35 (1.20–1.51)	< 0.001	1.29 (1.15–1.45)	< 0.001
Whether airbricks were present	Present	147 (70.3)	Reference	–	Reference	–
	Not present	469 (86.2)	1.23 (1.12–1.35)	< 0.001	0.96 (0.85–1.08)	0.49
Number of windows present	≥ 2 windows	114 (79.2)	Reference	–	Reference	–
	1 window	360 (80.5)	1.02 (0.92–1.12)	0.73	0.96 (0.87–1.05)	0.35
	No windows	142 (87.7)	1.11 (1.00–1.23)	0.05	0.98 (0.88–1.08)	0.65
Level of education	O level or beyond	134 (72.0)	Reference	–	Reference	–
	None or primary	482 (85.0)	1.18 (1.07–1.30)	0.001	1.13 (1.02–1.24)	0.02
Bed net ownership before enrolment	LLIN	472 (81.5)	Reference	–	Reference	–
	Untreated	88 (80.7)	0.99 (0.90–1.09)	0.85	0.96 (0.87–1.06)	0.45
	No bed net	56 (86.2)	1.06 (0.95–1.17)	0.30	1.01 (0.91–1.12)	0.87
Categories of gravidity	Multigravida	304 (79.6)	Reference	–	Reference	–
	Secundigravida	150 (80.2)	1.01 (0.92–1.10)	0.86	1.02 (0.94–1.11)	0.61
	Primigravida	162 (88.0)	1.11 (1.03–1.19)	0.007	1.10 (1.02–1.18)	0.009

*RR* risk ratio

^a^Adjusted for wealth index, type of house construction, level of education, bed net ownership before enrolment and categories of gravidity

^b^Modern houses defined as those with plaster or cement walls, metal or wooden roof, and closed eaves; all other houses defined as traditional

### Factors associated with parasitaemia during pregnancy following initiation of IPTp

Following the initiation of IPTp, a total of 3434 monthly routine assessments were conducted during pregnancy in 723 women, of which 15.4% were positive for malaria parasites by microscopy. Of 3412 blood smears assessed for parasitaemia by microscopy or qPCR, 43.0% were positive. Among the 30 women for whom household surveys were done but who had no routine assessments after initiation of IPTp, 25 were withdrawn before delivery and 5 were followed through delivery. The prevalence of microscopic parasitaemia was highest among women living in households in the lowest two wealth tertiles, however the association was significant only when comparing the middle tertile to the least poor tertile (aRR = 1.34, 95% CI 1.06–1.70, p = 0.02). Living in a house with no airbricks (aRR = 1.46, 95% CI 1.04–2.05, p = 0.03) and lower gravidity (aRR = 2.27, 95% CI 1.84–2.80, p < 0.001) were also associated with an increased risk of microscopic parasitaemia during pregnancy following the initiation of IPTp (Table 4). The strongest risk factor for microscopic parasitaemia during pregnancy was the use of IPTp with SP (aRR = 59.11, 95% CI 30.76–113.59, p < 0.001). For microscopic or sub-microscopic parasitaemia during pregnancy, low household wealth, living in a traditional house and having less education were associated with an increased risk. The strongest risk factors for microscopic or sub-microscopic parasitaemia during pregnancy were being primigravid compared to multigravid (aRR = 1.32, 95% CI 1.20–1.45, p < 0.001) and receiving IPTp with SP compared to DP (aRR = 3.13, 95% CI 2.84–3.46, p < 0.001) (Table 5).

**Table 4:** Factors associated with microscopic parasitaemia during pregnancy following initiation of IPTp

Variable	Categories	Parasitaemia^a^ n (%)	Univariate analysis		Multivariate analysis	
			RR (95% CI)	p-value	aRR^b^ (95% CI)	p-value
Wealth index categories	Least poor	135 (12.1)	Reference	–	Reference	–
	Middle	206 (17.8)	1.37 (1.01–1.87)	0.04	1.34 (1.06–1.70)	0.02
	Poorest	186 (16.0)	1.27 (0.93–1.73)	0.13	1.25 (0.97–1.62)	0.08
Type of house construction^c^	Modern	94 (12.1)	Reference	–	Reference	–
	Traditional	433 (16.3)	1.36 (0.99–1.85)	0.06	1.18 (0.91–1.53)	0.22
Whether airbricks were present	Present	107 (11.2)	Reference	–	Reference	–
	Not present	420 (16.9)	1.51 (1.12–2.03)	0.007	1.46 (1.04–2.05)	0.03
Number of windows present	≥ 2 windows	98 (14.7)	Reference	–	Reference	–
	1 window	326 (16.0)	1.09 (0.80–1.50)	0.58	0.95 (0.74–1.20)	0.65
	No windows	103 (14.1)	0.94 (0.63–1.39)	0.75	0.92 (0.68–1.24)	0.59
Level of education	O level or beyond	104 (12.6)	Reference	–	Reference	–
	None or primary	423 (16.2)	1.28 (0.95–1.73)	0.11	1.33 (1.03–1.72)	0.03
Categories of gravidity	Multigravida	202 (11.1)	Reference	–	Reference	–
	Secundigravida	107 (12.9)	1.18 (0.86–1.62)	0.30	1.44 (1.13–1.83)	0.003
	Primigravida	218 (27.5)	2.46 (1.89–3.20)	< 0.001	2.27 (1.84–2.80)	< 0.001
IPTp regimen	Monthly DP	9 (0.5)	Reference	–	Reference	–
	Monthly SP	518 (30.8)	59.71 (31.03–114.91)	< 0.001	59.11 (30.76–113.59)	< 0.001

*RR* risk ratio

^a^Measured at the time of routine visits done every 4 weeks during pregnancy (n = 3434)

^b^Adjusted for wealth index, type of house construction, level of education, categories of gravidity and IPTp regimen

^c^Modern houses defined as those with plaster or cement walls, metal or wooden roof, and closed eaves; all other houses defined as traditional

**Table 5:** Factors associated with microscopic or sub-microscopic parasitaemia during pregnancy following initiation of IPTp

Variable	Categories	Parasitaemia^a^ n (%)	Univariate analysis		Multivariate analysis	
			RR (95% CI)	p-value	aRR^b^ (95% CI)	p-value
Wealth index categories	Least poor	395 (35.7)	Reference	–	Reference	–
	Middle	539 (46.8)	1.28 (1.10–1.49)	0.001	1.25 (1.12–1.41)	< 0.001
	Poorest	534 (46.3)	1.28 (1.10–1.49)	0.001	1.24 (1.10–1.39)	< 0.001
Type of house construction^c^	Modern	285 (36.9)	Reference	–	Reference	–
	Traditional	1183 (44.8)	1.23 (1.06–1.44)	0.007	1.14 (1.01–1.28)	0.03
Whether airbricks were present	Present	345 (36.6)	Reference	–	Reference	–
	Not present	1123 (45.5)	1.26 (1.10–1.45)	0.001	1.16 (1.00–1.35)	0.05
Number of windows present	≥ 2 windows	274 (41.3)	Reference	–	Reference	–
	1 window	885 (43.9)	1.08 (0.93–1.26)	0.32	0.99 (0.88–1.11)	0.85
	No windows	309 (42.3)	1.04 (0.87–1.25)	0.64	1.00 (0.87–1.14)	0.98
Level of education	O level or beyond	301 (36.6)	Reference	–	Reference	–
	None or primary	1167 (45.1)	1.23 (1.06–1.43)	0.006	1.19 (1.06–1.34)	0.002
Categories of gravidity	Multigravida	705 (39.1)	Reference	–	Reference	–
	Secundigravida	337 (41.0)	1.05 (0.90–1.21)	0.55	1.17 (1.05–1.31)	0.006
	Primigravida	426 (54.1)	1.38 (1.22–1.57)	< 0.001	1.32 (1.20–1.45)	< 0.001
IPTp regimen	Monthly DP	367 (21.1)	Reference	–	Reference	–
	Monthly SP	1101 (65.9)	3.11 (2.81–3.45)	< 0.001	3.13 (2.84–3.46)	< 0.001

*RR* risk ratio

^a^Measured at the time of routine visits done every 4 weeks during pregnancy (n = 3412)

^b^Adjusted for wealth index, type of house construction, level of education, categories of gravidity and IPTp regimen

^c^Modern houses defined as those with plaster or cement walls, metal or wooden roof, and closed eaves; all other houses defined as traditional

### Factors associated with placental malaria

A total of 44.6% of 653 women had evidence of placental malaria by histopathology. Although traditional house construction and the absence of airbricks were risk factors for placental malaria in univariate analysis, these associations were not significant in multivariate analysis. The only factors associated with increased risk of placental malaria in multivariate analysis were lower gravidity (aRR = 2.87, 95% CI 2.39–3.45, p < 0.001) and IPTp with SP compared to DP (aRR = 2.13, 95% CI 1.79–2.53, p < 0.001) (Table 6).

**Table 6:** Factors associated with placental malaria

Variable	Categories	Placental malaria, n (%)	Univariate analysis		Multivariate analysis	
			RR (95% CI)	p-value	aRR^a^ (95% CI)	p-value
Wealth index categories	Least poor	83 (39.5)	Reference	–	Reference	–
	Middle	99 (44.8)	1.13 (0.91–1.42)	0.27	1.02 (0.84–1.24)	0.83
	Poorest	109 (49.1)	1.24 (1.00–1.54)	0.05	1.14 (0.94–1.38)	0.18
Type of house construction^b^	Modern	53 (35.8)	Reference	–	Reference	–
	Traditional	238 (47.1)	1.32 (1.04–1.66)	0.02	1.19 (0.96–1.48)	0.10
Whether airbricks were present	Present	64 (35.6)	Reference	–	Reference	–
	Not present	227 (48.0)	1.35 (1.09–1.68)	0.007	1.06 (0.81–1.39)	0.67
Number of windows present	≥ 2 windows	53 (40.2)	Reference	–	Reference	–
	1 window	172 (45.1)	1.12 (0.89–1.42)	0.33	0.99 (0.80–1.22)	0.93
	No windows	66 (47.1)	1.17 (0.89–1.54)	0.25	1.04 (0.81–1.33)	0.76
Level of education	O level or beyond	65 (41.9)	Reference	–	Reference	–
	None or primary	226 (45.4)	1.08 (0.88–1.33)	0.46	1.16 (0.95–1.41)	0.14
Categories of gravidity	Multigravida	94 (27.3)	Reference	–	Reference	–
	Secundigravida	72 (47.1)	1.72 (1.35–2.19)	< 0.001	1.85 (1.48–2.31)	< 0.001
	Primigravida	125 (80.1)	2.93 (2.43–3.54)	< 0.001	2.87 (2.39–3.45)	< 0.001
IPTp regimen	Monthly DP	94 (28.4)	Reference	–	Reference	–
	Monthly SP	197 (61.2)	2.15 (1.78–2.61)	< 0.001	2.13 (1.79–2.53)	< 0.001

*RR* risk ratio

^a^Adjusted for wealth index, type of house construction, level of education, categories of gravidity and IPTp regimen

^b^Modern houses defined as those with plaster or cement walls, metal or wooden roof, and closed eaves; all other houses defined as traditional

## Discussion

This study investigated the association between maternal and household factors and malaria in pregnancy in a rural, high transmission setting in Uganda. Gravidity was consistently and strongly associated with malaria throughout pregnancy, with primigravid women having an 84% higher risk of microscopic parasitaemia at enrolment, more than double the risk of microscopic parasitaemia following the initiation of IPTp and nearly three times the risk of placental malaria, compared to multigravid women. Similarly, IPTp with SP was associated with nearly a 60-fold higher risk of malaria parasitaemia and doubled risk of placental malaria, compared to DP. Of the household and other maternal factors assessed, belonging to the poorest households, living in traditional houses and having only primary or no education were associated with an increased risk of malaria in pregnancy, compared to the least poor households, modern houses and having at least O-level education. To the knowledge of the authors, this is the first study to evaluate household risk factors for malaria in pregnancy in Uganda.

Gravidity is a well-established risk factor for malaria in pregnancy in high-transmission areas, where multigravid women have a lower risk of infection due to the acquisition of immunity through consecutive pregnancies [2]. In contrast, as transmission declines the burden of malaria shifts from paucigravidae towards all pregnant women. The present finding that primigravid women were at a higher risk of malaria parasitemia and placental malaria than multigravid women, despite high coverage with IPTp, is consistent with the gravidity-related pattern observed in other high-transmission settings [26]. A strong association between IPTp regimen and malaria infection in pregnancy and placental malaria was also observed, as well as a lower prevalence of malaria infection following the initiation of IPTp. Indeed, the parent study found that DP provided almost complete protection against microscopic parasitaemia [23] and an earlier randomized controlled trial in neighbouring Tororo found that IPTp with DP reduced malaria in pregnancy more than IPTp with SP, although DP was not associated with a lower risk of malaria in infancy [27]. IPTp with SP is a primary intervention recommended by the WHO for preventing malaria in pregnancy. However, among 33 African countries that reported on IPTp coverage levels in 2017, only an estimated 22% of eligible pregnant women received the recommended three or more doses [28]. The present study reiterates the need to maintain high effective coverage with IPTp to protect women in all pregnancies, as well as identifying and using the most efficacious regimen, such as DP, where possible.

Malaria in pregnancy remains a major public health challenge in Uganda and these findings suggest that within communities the risk is highest among the poorest women. A study in neighbouring Tororo district found an association between malaria in children and socioeconomic position, a relationship hypothesized to be explained by causal pathways that include access to and uptake of interventions and treatment-seeking behaviour, nutrition, housing conditions and education [29]. In the present study, modern housing, higher education levels and bed net ownership at enrolment were all associated with greater wealth. Importantly, an association between malaria and poverty was detected even after controlling for IPTp regimen, indicating that social and economic conditions may be relevant even where protective interventions are in place. This suggests a concerning cycle in which children born to less socioeconomically advantaged mothers may have worse health outcomes at birth and subsequently in later life, than those born to more advantaged mothers.

In this study, traditional housing and lower educational attainment were also associated with an increased risk of malaria infection during pregnancy compared to modern housing and more education. Housing quality can affect malaria risk through its effect on house entry on the primary malaria vector *Anopheles gambiae,* which mainly bites people indoors at night-time [30]. The relationship between house design and malaria in the general population has been well-studied [31], but few studies have evaluated its association with malaria in pregnancy. A recent meta-analysis, however, revealed a strong linear relationship between malaria infection in children and in pregnant women [26], so these groups plausibly have overlapping risk factors. Meanwhile, education may offer a protective effect against malaria through increased knowledge of the disease and use of preventive interventions such as LLINs. Studies elsewhere have shown women’s education status to be associated with the risk of malaria among children [32], including in Uganda [33]. Supplementary approaches to prevent malaria in pregnancy, such as housing improvements and education initiatives, may therefore merit consideration.

This study had several limitations. First, self-report was used to measure the variables used to calculate the wealth index and educational attainment. The wealth index is also an imperfect metric and influenced by the variables included. Indeed, the observed relationship between housing, education and malaria is likely to have been affected by residual confounding by household wealth. Second, since the study was a secondary analysis of data from a randomized controlled trial, it was not possible to assess a complete range of risk factors for malaria in pregnancy, such as maternal marital status and occupation, as well as certain household characteristics such as screening of airbricks. Third, the findings of this study may not be generalizable to other settings with different malaria transmission profiles. Fourth, maternal age was not included in the analysis, since this was highly co-linear with gravidity, but younger maternal age (especially adolescence) is a known risk factor for malaria in pregnancy due to the lack of age-associated immunity [2]. Finally, the observed associations between household and maternal factors and malaria risk are not evidence of causality [34]. For example, it was found that the poorest women had a 24% greater risk of microscopic or submicroscopic parasitaemia than the least poor, after controlling for factors including IPTp regimen. However, this may plausibly be explained by the direct and indirect costs of malaria contributing to poverty, lower educational attainment and the use of more affordable building materials. Nonetheless, to the knowledge of the authors, this study provides the first evaluation of household risk factors for malaria in pregnancy among Ugandan women.

## Conclusions

This study provides evidence that the risk of malaria in pregnancy in Uganda is highest among primigravid women and those belonging to the poorest households, living in traditional homes and with the least education. Alongside efforts to maintain high coverage with IPTp, LLINs and prompt and effective diagnosis and treatment, housing improvements and education initiatives could be explored as supplementary approaches to reduce malaria in pregnancy.

## Abbreviations

DP: dihydroartemisinin–piperaquine
IDRC: Infectious Diseases Research Collaboration
IPTp: intermittent preventive treatment for malaria during pregnancy
LLIN: long-lasting insecticidal net
MOH: Ministry of Health
NMCP: National Malaria Control Programme
NICHD: National Institute of Child and Human Development
qPCR: quantitative polymerase chain reaction
UCSF: University of California San Francisco
SP: sulfadoxine–pyrimethamine
WHO: World Health Organization
